# Recombinant viral capsid protein VP1 suppresses lung cancer metastasis by inhibiting COX-2/PGE2 and MIG-7

**DOI:** 10.18632/oncotarget.2040

**Published:** 2014-05-29

**Authors:** Ming-Yi Ho, Shao-Wen Hung, Chi-Ming Liang, Shu-Mei Liang

**Affiliations:** ^1^ Genomics Research Center, Academia Sinica, Taipei, Taiwan, ROC; ^2^ Agricultural Biotechnology Research Center, Academia Sinica, Taipei, Taiwan, ROC; ^3^ Institute of Biological Chemistry, Academia Sinica, Taipei, Taiwan, ROC

**Keywords:** Migration inducting gene-7 (MIG-7), Cyclooxygenase-2 (COX-2), Integrin-linked kinase (ILK), rVP1, Cancer metastasis, EMT

## Abstract

Recombinant capsid protein VP1 (rVP1) of foot-and-mouth disease virus binds to integrins to modulate Akt/GSK3-β signaling and suppress migration/invasion and metastasis of cancer cells, but the underlying molecular mechanism is unclear. Here, we showed that the rVP1-mediated inhibition of Akt/GSK3-β signaling and cell migration/invasion was accompanied by downregulation in phosphatidylinositol (3,4,5)-triphosphate (PIP3), integrin-linked kinase (ILK) and IKK/NF-κB signaling as well as suppression of COX-2/PGE2 and MIG-7. Addition of PIP3 or overexpression of ILK reversed the rVP1-induced inhibition of IKK/NF-κB signaling, COX-2 and MIG-7. The rVP1-mediated downregulation of COX-2/PGE2 and MIG-7 led to not only attenuation of epithelial-mesenchymal transition, MMP2 activity and invasion of lung cancer cells *in vitro* but also decreased tumor growth and metastasis of lung cancer in xenograft mice. Moreover, downregulation of COX-2/PGE2 and MIG-7 significantly prolonged the overall and disease-free survival of lung cancer-bearing mice. These results suggest that rVP1 inhibits cancer invasion/metastasis, partly if not mainly, via downregulating integrin/PI3K/Akt, ILK and IKK/NF-κB signaling to suppress expression of COX-2/PGE2 and MIG-7.

## INTRODUCTION

Lung cancer is a leading cause of cancer-related death worldwide [1, 2]. Metastasis is the primary cause of lung cancer treatment failure and mortality [1, 3]. Understanding the cellular mediators that contribute to the invasion and metastasis of lung cancer and the development of novel therapeutic agents targeting these mediators are urgently needed.

One of the potential mediators of metastasis is cyclooxygenase-2 (COX-2), the inducible isoform of COX. COX-2 is frequently found in early and advanced lung cancer tissues and is associated with poor prognosis [4-7]. Elevation of tumor COX-2 increases the level of its metabolite prostaglandin E2 (PGE2) that is a ligand of G protein-coupled receptors, such as EP1, EP2, EP3, and EP4. COX-2/PGE2 stimulates phosphatidylinositol 3-kinase/protein kinase B (PI3K/Akt) and extracellular signal-regulated kinase 1/2 (ERK1/2) signaling to induce tumor angiogenesis, cancer motility and invasiveness [8-11]. Recently COX-2/PGE2 has also been found to induce migration inducing gene-7 (MIG-7) protein that sustains the activation of PI3K/Akt/glycogen synthase kinase-3 β (GSK-3β) signaling via decreasing the activity of protein phosphatase 2A (PP2A) to increase lung cancer invasion/metastasis [12].

Recombinant DNA-derived VP1 (rVP1) of foot-and-mouth disease virus (FMDV) has previously been found to induce apoptosis of human cancer cell lines MCF-7, PC-3 and 22Rv1 via modulation of the integrin/Akt signaling pathway [13]. Recently, rVP1 has also been shown to suppress progression of hepatocellular carcinoma [14] and invasion of SKOV3 ovarian adenocarcinoma cells as well as SiHa and Caski cervical carcinoma cells [15, 16]. However, the molecular mechanisms underlying the inhibition of cancer invasion/metastasis by rVP1 remain largely unexamined.

In this study, we explored whether rVP1 has any effect on COX-2/PGE2 and MIG-7. Our results showed that rVP1 suppressed epithelial-mesenchymal transition (EMT), migration/invasion and metastasis of human lung cancer cells. Its mechanism of action involved modulation of PIP3 and ILK in the lipid rafts as well as suppression of IKK/NF-κB, COX-2/PGE2 and MIG-7. The role of rVP1-mediated decrease of COX-2/PGE2 and MIG-7 in inhibiting tumor growth and metastasis of lung adenocarcinoma was further substantiated by analyzing COX-2/PGE2 and MIG-7 in the lung cancer xenograft mice with or without rVP1 treatment.

## RESULTS

### rVP1 suppresses EMT, MMP-2 and migration/invasion of human lung cancer cells

**Figure 1:**
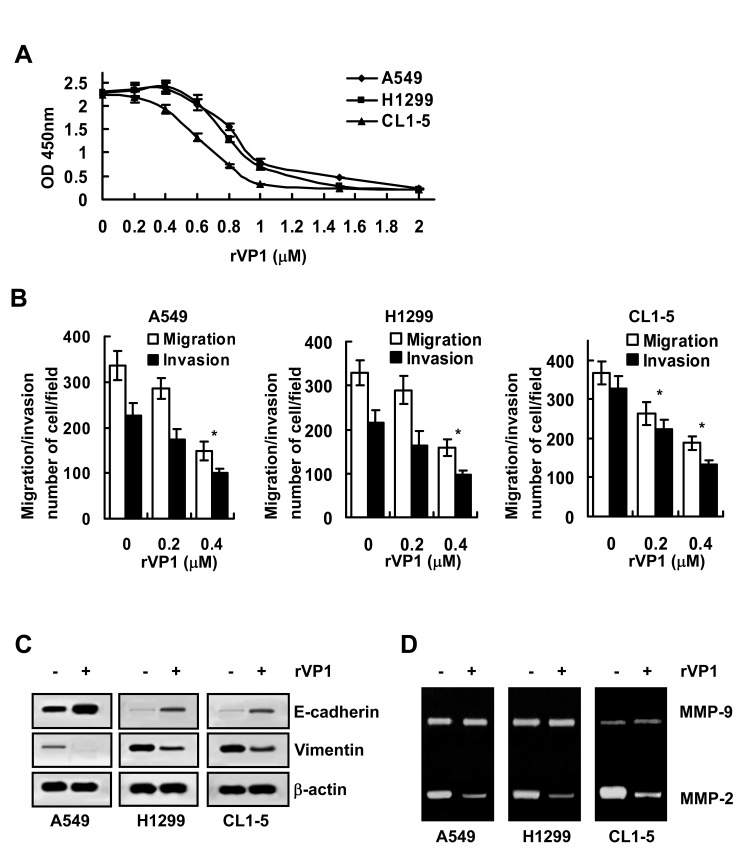
rVP1 suppresses the migration/invasion of lung cancer cells *in vitro* Lung cancer cells (A549, H1299 and CL1-5; 2 × 10^5^/mL) were treated with 0.4 μM rVP1 in 0.5% FBS medium for 24 h, unless specified otherwise. (A) Lung cancer cells were treated with serial concentrations of rVP1 (0–2 μM) as indicated. Cell proliferation was measured by WST-1 assay *in vitro*. Data represent means ± SD of three independent experiments. (B) The migration and invasion capability of cells with or without rVP1 treatment were measured by transwell migration assay. Data represent means ± SD of three independent experiments; ^*^P < 0.05 by t-test. (C) The expression level of epithelial cell marker E-cadherin and mesenchymal cell marker vimentin were analyzed by immunoblotting. β-actin was used as a loading control. Blots are representative of three independent experiments. (D) The MMP-2 and MMP-9 enzyme activity in the cell-cultured medium was analyzed by a gelatin zymography assay. Data are representative of three independent experiments.

We previously showed that rVP1 suppresses growth and migration/invasion of ovarian and cervical cancer [15, 16]. To investigate whether rVP1 inhibits lung cancer cells in a similar manner, three human lung cancer cell lines, A549, H1299 and CL1-5 were treated with rVP1. Our results showed that rVP1 inhibited the cell viability of A549, H1299 and CL1-5 in a concentration-dependent manner (Figure 1A) with IC_50_ values of 0.93, 0.88 and 0.68 μM, respectively. Interestingly, even at concentrations (0.2 μM - 0.4 μM) that caused only minimal cell death (Figure 1A), rVP1 dramatically suppressed migration and invasion of A549, H1299 and CL1-5 cells (Figure 1B).

Since the downregulation of epithelial cell marker E-cadherin and a concomitant increase in the expression of the mesenchymal cell marker vimentin correlate with enhancement in EMT and migration/invasion of cancer cells [17], we determined the effect of rVP1 on the expression level of E-cadherin and vimentin in lung cancer cells. Immunoblotting analysis revealed that treatment with rVP1 (0.4 μM) for 24 h attenuated EMT by upregulating E-cadherin and downregulating vimentin expression levels in A549, H1299 and CL1-5 lung cancer cell lines (Figure 1C).

As MMP activity is positively associated with enhanced cellular invasion [18], we next evaluated the effect of rVP1 on MMP activity of lung cancer cells. Gelatin zymographic analysis showed that the MMP-2 activity in A549, H1299 and CL1-5 cells was reduced by rVP1 treatment (Figure 1D). These results thus demonstrated that rVP1 decreases EMT and MMP-2 activity to suppress the migration and invasion of lung cancer cells.

### rVP1 downregulates integrin β1/Akt, COX-2/PGE2 and MIG-7 to suppress lung cancer cell migration/invasion

**Figure 2:**
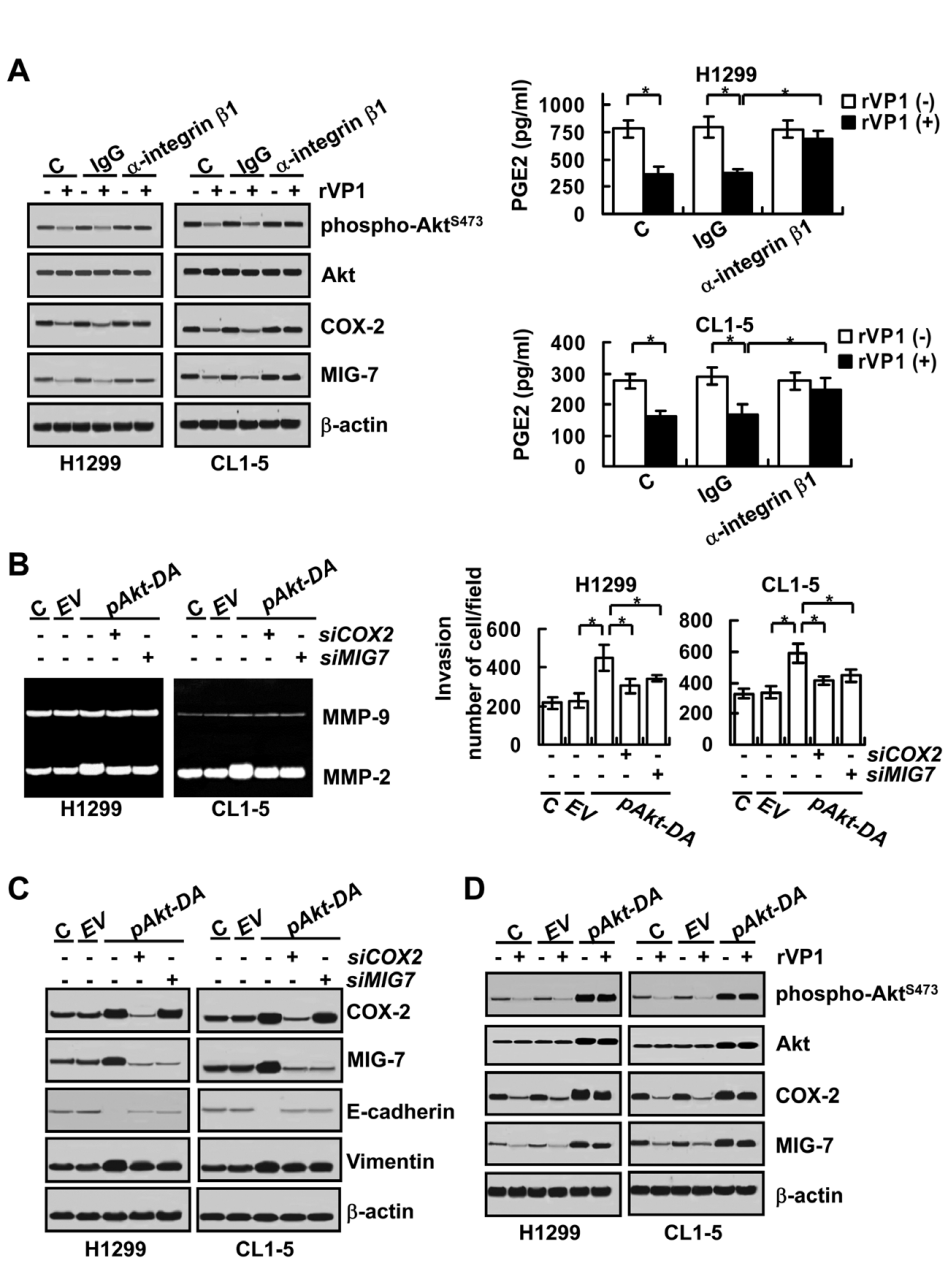
rVP1 binds to integrin to inhibit COX-2 and MIG-7 and decrease lung cancer cell invasion Lung cancer cells (H1299 and CL1-5; 2 × 10^5^/mL) were treated with control IgG or anti-integrin β1 antibodies (2 μg/ml) for 30 min followed by 0.4 μM rVP1 for 24 h in 0.5% FBS medium. Proteins were determined by immunoblotting. β-actin was used as a loading control. Blots are representative of three independent experiments. Cell-conditioned media were examined for PGE2 by PGE2 EIA kit (right panels). Data represent means ± SD of three independent experiments; *P < 0.05 by t-test. (B and C) Parental lung cancer cells (H1299 and CL1-5; C) were transfected with empty vector (EV), dominant-active Akt (*pAkt-DA*) plasmids, control scrambled siRNAs (-*siCOX2* and -*siMIG7*), COX2 siRNAs (+*siCOX2*) or MIG7 siRNAs (+*siMIG7*) for 48 h. (D) Cells (H1299 and CL1-5; C) transfected with empty vector (EV) or dominant-active Akt (*pAkt-DA*) plasmids for 48 h were treated with 0.4 μM rVP1 for 24 h in 0.5% FBS medium. Immunoblotting was used for determining proteins, zymography assay for MMP activity and transwell assay for cell invasion as described in Materials and Methods. Blots are representative of three independent experiments. Data represent means ± SD of three independent experiments; *P < 0.05 by t-test.

rVP1 suppresses migration/invasion of cervical cancer cells via downregulating the Akt signaling pathway through integrin β1 [15]. Since inhibition of integrin β1 signaling has been reported to attenuate COX-2 and MIG-7 levels [19, 20], we examined whether rVP1 suppresses expression of COX-2 and MIG-7 in human lung cancer cells through the integrin β1/Akt pathway. Our results showed that phospho-Akt^S473^, COX-2, PGE2 and MIG-7 were decreased in lung cancer cells treated with rVP1 (0.4 μM) for 24 h (Figure 2A). The rVP-1-mediated reduction of Akt^S473^ phosphorylation as well as COX-2, PGE2 and MIG-7 expression was reversed by anti-integrin β1 antibodies, but not by the control immunoglobulin G (IgG) (Figure 2A). Constitutive phosphorylation of Akt at serine 473 (Akt^S473^) by transfecting lung cancer cells with dominant active Akt plasmid (*pAkt-DA*) increased the EMT, MMP2 activity and invasion ability of cancer cells and increased expression of COX-2 and MIG-7 (Figure 2B and 2C). These effects of constitutively active Akt were reversed by knockdown of COX-2 or MIG-7 with transfection of COX-2 siRNA (*siCOX-2*) or MIG-7 siRNA (*siMIG-7*) (Figure 2B and C). As anticipated, the constitutively active-Akt-mediated COX-2 and MIG-7 expression was not attenuated by rVP1 (0.4 μM) (Figure 2D). These results suggest that rVP1 inhibits EMT, MMP2 activity and the invasion of lung cancer cells via downregulating phosphorylated Akt as well as suppression of COX-2 and MIG-7 expression.

### PIP3 and ILK play essential roles in rVP1-mediated downregulation of IKK/NF-κB signaling and COX-2

**Figure 3:**
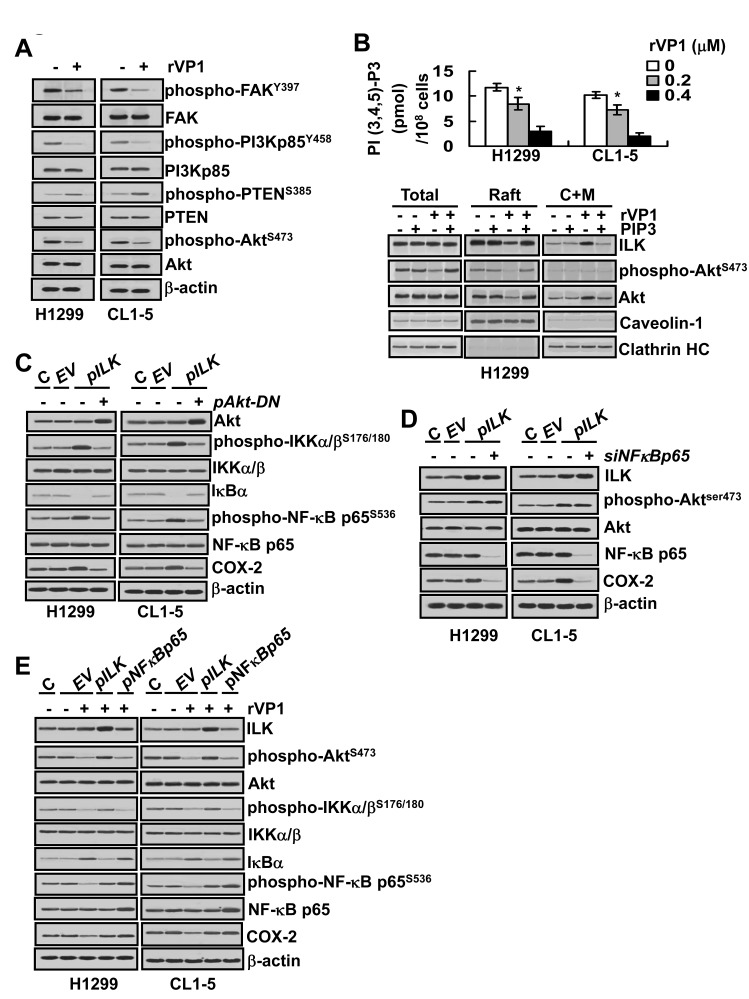
rVP1 decreases COX-2 via modulating PIP3 and IKK/NF-B signaling in lung cancer cells (A) H1299 and CL1-5 cells were treated with or without 0.4 μM rVP1 for 24 h in 0.5% FBS medium. Proteins were determined by immunoblotting. β–actin was used as a loading control. (B) After treatment with 0.2 μM or 0.4 μM rVP1 for 24 h in 0.5% FBS medium, cellular phospholipids were extracted and PIP3 levels determined by ELISA (upper panel). Data represent means ± SD of three independent experiments; ^*^P < 0.05 by t-test. H1299 and CL1-5 cells were treated with 0.4 μM rVP1, 5 μM PIP3 or rVP1 plus PIP3 as indicated for 24 h in 0.5% FBS medium. Proteins in the membrane raft or cytosol plus non-raft membrane (C+M) fractions were determined by immunoblotting. Caveolin-1 served as a membrane raft marker and clathrin-HC served as a membrane non-raft marker (lower panel). (C and D) Parental lung cancer cells (H1299 and CL1-5; C) were transfected with empty vector (EV and -*pAkt-DN*), pILK plasmids (*pILK*), dominant-negative Akt (+*pAkt-DN*) plasmids, control scrambled siRNAs (-*siNFκBp65*), or NFκBp65 siRNA (+*siNFκBp65*) for 48 h. (E) Parental cells (H1299 and CL1-5; C) transfected with empty vector (EV), *pILK* or *pNF-κBp65* for 48 h were treated with 0.4 μM rVP1 for 24 h in 0.5% FBS medium. Proteins were examined by immunoblotting. Blots are representative of three independent experiments.

Phosphatidylinositol 3-kinase (PI3K) phosphorylates phosphatidylinositol (4,5)-biphosphate (PIP2) to generate phosphatidylinositol (3,4,5)-triphosphate (PIP3) that can bind to the pleckstrin-homology domains of integrin-linked kinase (ILK) and Akt to increase the attachment of ILK and Akt to lipid rafts, leading to the activation of Akt signaling pathways [21-23]. To elucidate the mechanism by which rVP1 acts on the integrin β1/Akt signaling of lung cancer cells, we examined the effects of rVP1 on integrin downstream targets, PI3K, PIP3, ILK and Akt. Our results showed that rVP1 increased phosphorylation of phosphatase PTEN accompanied by a decrease in phosphorylation of focal adhesion kinase (FAK), a molecule upstream of PI3K, as well as PI3K-p85^Y458^ and Akt^S473^ (Figure 3A). Further studies showed that rVP1 decreased PIP3 and prevented accumulation of ILK, Akt and phospho-Akt^S473^ in the raft domain (Figure 3B). Addition of PIP3 reversed the inhibitory effect of rVP1 on the level of ILK, Akt and phospho-Akt^S473^ in the lipid rafts (Figure 3B). These results demonstrated that rVP1 decreases phosphorylation of FAK, PI3K and PIP3 leading to a decline in ILK and phospho-Akt^S473^ in the raft domains.

To further understand how rVP1 could reduce expression of COX-2, we next examined whether ILK or the IKK/NF-κB pathway plays any role in the inhibitory effect of rVP1 on COX-2 expression in lung cancer cells. We found that overexpression of ILK by transfection of ILK plasmid (*pILK*) in H1299 and CL1-5 lung cancer cells increased phosphorylation of Akt^S473^, IKKα/β^S176/180^ and NF-κB p65^S536^ as well as degradation of IκB, which were positively correlated with elevation of COX-2 level (Figure 3C and 3D). Transduction of inactive Akt by transfection of Akt-DN plasmid (*pAkt-DN*) blocked the ILK-mediated induction of IKKα/β-IκB-NF-κB signaling and expression of COX-2 (Figure 3C). Even though knockdown of NF-κB p65 by transfection of NF-κB p65 siRNA (+*siNFκBp65*) did not affect phosphorylation of Akt^S473^, it also suppressed the ILK-induced COX-2 expression (Figure 3D). Overexpression of ILK or NF-κB p65 by transfection with *pILK* or *pNFκBp65* blocked the inhibition of COX-2 expression by rVP1 (Figure 3E). Taken together, these results indicate that rVP1 inhibits integrin/FAK/PI3K to decrease the level of PIP3 which in turn modulates ILK level in the lipid rafts to regulate IKK/NF-κB pathway signaling and COX-2 expression.

### rVP-1 suppresses COX-2 and MIG-7 to mitigate EMT and migration/invasion of lung cancer cells

**Figure 4:**
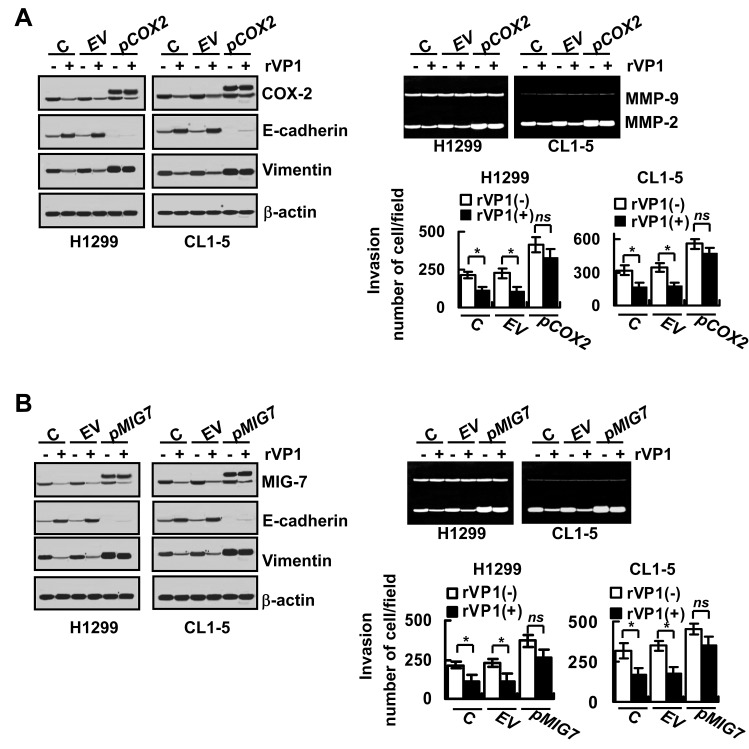
Overexpression of COX-2 and MIG-7 reverses rVP1-mediated inhibition on EMT, MMP-2 activity and lung cancer cell invasion (A) Parental cells (H1299 and CL1-5; C) transfected with empty vector (EV) and pCOX2 plasmids (*pCOX2*) for 48 h were treated with 0.4 μM rVP1 as indicated for 24 h in 0.5% FBS medium. (B) Cells transfected with empty vector (EV) and pMIG7 plasmids (*pMIG7*) for 48 h were treated with 0.4 μM rVP1 as indicated for 24 h in 0.5% FBS medium. Cells were examined by immunoblotting for protein expression, zymography for MMP activity and transwell assay for cell invasion. Blots are representative of three independent experiments. Data represent means ± SD of three independent experiments; *P < 0.05 by t-test.

We recently showed that COX-2/PGE2 activates EP4 to enhance Akt and GSK-3β phosphorylation and β-catenin/LEF/TCF signaling leading to MIG-7 upregulation [12]. To investigate whether rVP1-mediated decrease of COX-2/PGE2 (and/or MIG-7) is functionally associated with rVP1/integrin β1/Akt-mediated reduction in EMT, MMP2 and invasion of lung cancer cells, we transfected lung cancer cell lines (H1299 and CL1-5) with COX-2 or MIG-7 plasmids (*pCOX2* or *pMIG7*) to overexpress COX-2 or MIG-7 protein. We found that transfection with *pCOX2* increased COX-2 together with MIG-7 protein, EMT, MMP-2 activity and invasion of lung cancer cells (Figure 4A and Supplementary Figure 1). PGE2 treatment also increased MIG-7 protein, EMT, MMP-2 activity and invasion of lung cancer cells (Supplementary Figure 1). MIG-7 knockdown (+*siMIG7*) attenuated the effects of COX-2 overexpression and PGE2 treatment on MIG-7, EMT, MMP-2 and the invasion of the cells, but did not affect expression level of COX-2 (Supplementary Figure 1). The rVP-1-mediated suppression of EMT as shown by induction of E-cadherin expression and reduction of vimentin could be reversed in cancer cells by transduction of *pCOX-2* or *pMIG-7* (Figure 4A and 4B). In addition, overexpression of COX-2 and MIG-7 reversed the inhibition of MMP2 enzyme activity and invasion ability of H1299 and CL1-5 cells by rVP1 (Figure 4A and 4B). Collectively, these results suggest that rVP1 modulates integrin β1/Akt signaling to downregulate COX-2/PGE2 that then attenuates MIG-7 protein level resulting in inhibition of the EMT, MMP-2 activity and invasion of lung cancer cells.

### rVP-1 reduces COX-2 and MIG-7 and suppresses metastasis and lethal effects of lung cancer cells *in vivo*

**Figure 5:**
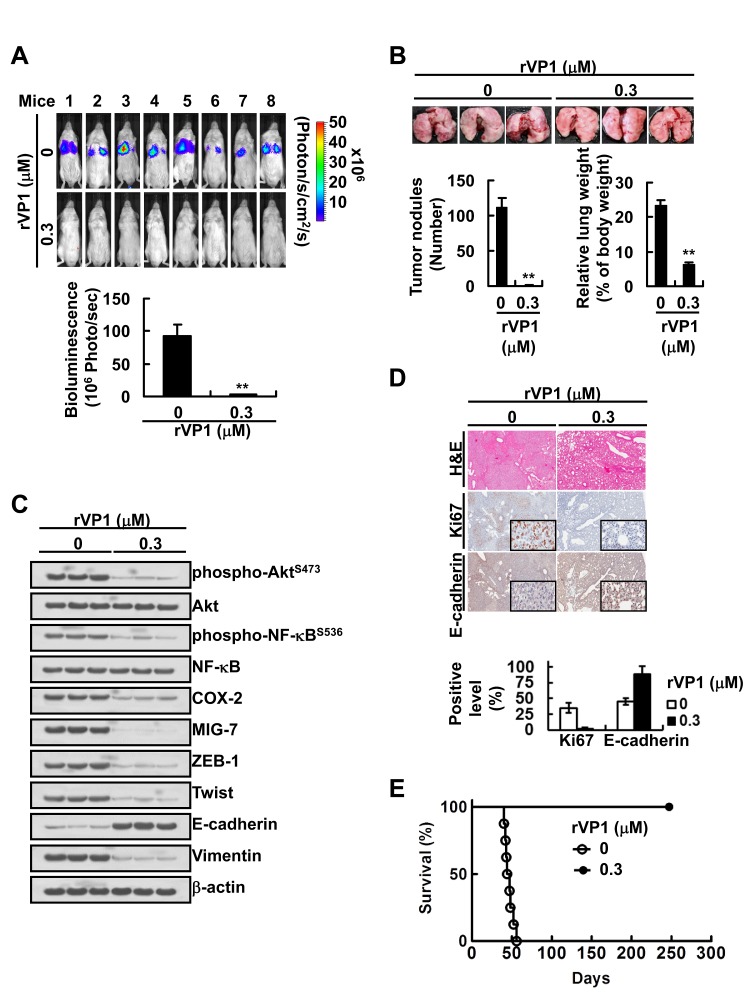
Treatment of lung cancer cells with low concentration of rVP1 decreases their tumor forming capability and lethality *in vivo* CL1-5^GL^ cells stably expressing green fluorescent protein and luciferase (GL) were generated as described in Materials and Methods. CL1-5^GL^ cells were pretreated with or without 0.3 μM rVP1 for 24 h in 0.5% FBS medium and were implanted into SCID mice (1 × 10^6^ cells/50 μL/mouse; *n* = 11) *via* tail-vein injection. (A and B) The whole bodies of mice underwent bioluminescent imaging 5 weeks after inoculation and the lungs of mice were dissected from the surrounding tissue to measure weight and count tumor nodules. Data represent means SD of at least 3 mice of each group; **P < 0.01 by t-test. (C and D) The murine lung sections were analyzed after H&E (at ×40 magnification) and immunohistochemistry staining (at ×40 magnification; insets ×400 magnification) and cell lysates were extracted and analyzed by immunoblotting. Blots of three tissue samples of each group are representative of three independent experiments. (E) Percentage of survival of mice inoculated with rVP1-pretreated CL1-5^GL^ cells was compared with that of mice inoculated with cells without rVP1 pretreatment; *P* < 0.0001, *n* = 6 by t-test.

To examine whether rVP1 inhibits lung cancer metastasis via downregulating COX-2 and MIG-7 *in vivo*, we generated CL1-5^GL^ cells stably expressing green fluorescent protein and luciferase (GL). Some CL1-5^GL^ cells were pretreated for 24 h with rVP1 at such a low concentration (0.3 μM) that inhibited cell invasion (Supplementary Figure 2A) but not cell proliferation (Supplementary Figure 2B). The CL1-5^GL^ cells with or without rVP1 pretreatment, respectively, were then implanted into two groups of SCID mice *via* tail-vein injection. After implantation, a significant reduction of tumor nodules in the lung was observed in mice injected with rVP1-pretreated-CL1-5^GL^ cells (Figure 5A and 5B) as compared to those injected with CL1-5^GL^ cells that were not pretreated with rVP1. Analysis of lung tissue lysates showed that mice implanted with rVP1-pretreated CL1-5^GL^ had lower EMT, phospho-Akt^S473^, phospho-NF-κB^S536^, COX-2 and MIG-7 than those implanted with CL1-5^GL^ without rVP1 pretreatment (Figure 5C). Histopathologic examination showed that there was more downregulation of Ki67 and upregulation of E-cadherin in in the rVP1-pretreated group than in the control group (Figure 5D). Moreover, all the mice implanted with CL1-5^GL^ without rVP1 pretreatment died on or before day 50 post-inoculation; whereas all the mice implanted with rVP1-pretreated-CL1-5^GL^ cells were disease free on day 250.

**Figure 6:**
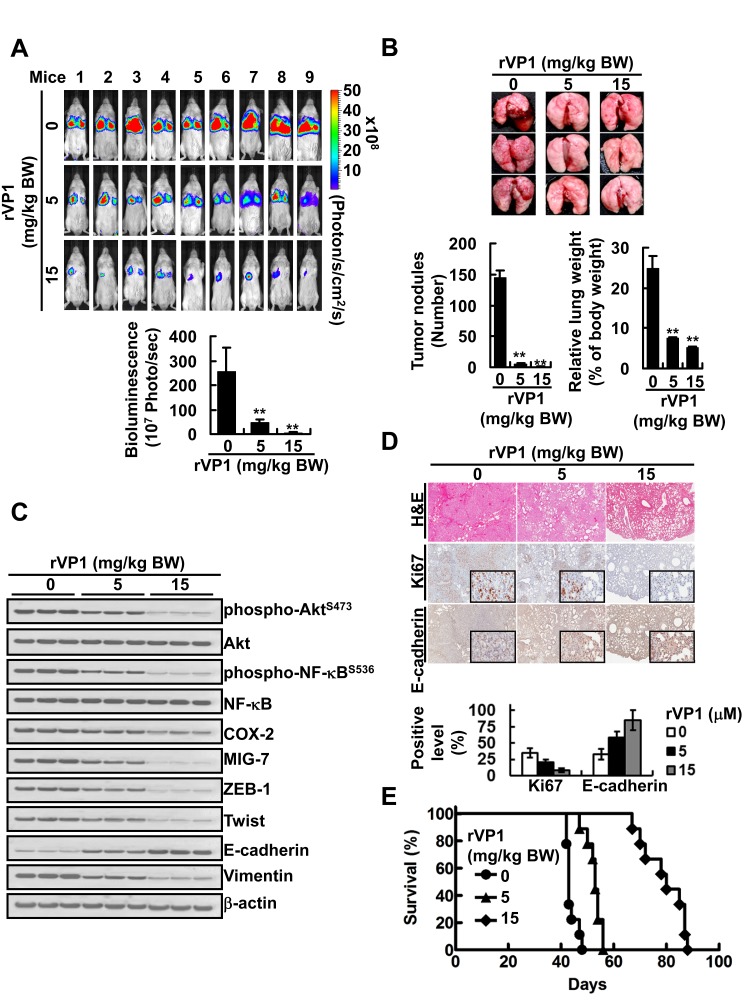
rVP1 decreases COX-2 and MIG-7 and suppresses lung cancer metastasis in xenograft mice CL1-5^GL^ stable cells were injected into SCID mice tail vein (*n* = 12). One week after implantation, rVP1 (5 or 15 mg/kg body weight) was administered *via* tail-vein injection three times *per* week for four weeks. (A and B) The whole bodies of mice underwent bioluminescent imaging and the lungs of mice were dissected from the surrounding tissue to measure weight and count tumor nodules. Data represent means ± SD of at least 3 mice from each group; **P < 0.01 by t-test. (C and D) The murine lung sections were analyzed after H&E (at ×40 magnification) and immunohistochemistry staining (at ×40 magnification; insets ×400 magnification) and cell lysates were extracted and analyzed by immunoblotting. Blots of three tissue samples of each group are representative of three independent experiments. (E) Percentage of survival of CL1-5^GL^-bearing mice treated with vehicle was compared with those treated with rVP1. Without rVP1 treatment t_50_ = 42 day, 5 and 15 mg/kg body weight of rVP1 treatment t_50_ = 55 and 78 day, respectively; *n* = 9; *P* < 0.001.

To further investigate the *in vivo* effect of rVP1, instead of pretreating CL1-5^GL^ cells with rVP1, we implanted the cancer cells into SCID mice and one week later start administering rVP1 (5 or 15 mg/kg body weight) intravenously *via* the tail vein three times per week for 4 weeks. Although not as effective as pretreatment, rVP1 still significantly reduced tumor growth in the lungs of CL1-5^GL^-bearing mice as compared to those treated with control vehicle (Figure 6A and 6B). Analysis of tumor lysates obtained from the vehicle- and rVP1-treated mice showed that the levels of EMT, phospho-Akt^S473^, phospho-NF-κB^S536^, COX-2 and MIG-7 in tumors from the rVP1-treated mice were lower than those in tumors from the vehicle-treated mice (Figure 6C). Histological analysis of lungs revealed that there was less dissemination of tumor cells in lung tissue sections from rVP1-treated mice whereas many more cancer cells were found in vehicle-treated mice (Figure 6D) as indicated by Ki67 level. Comparison of the survival curve of rVP1-treated mice with that of vehicle-treated mice showed that rVP1 treatment significantly prolonged the survival of tumor-bearing mice (Figure 6E). Taken together, these results suggest that rVP1-mediated downregulation of COX-2 and MIG-7 suppressed EMT and the metastatic capability of human lung cancer cells, and therefore prolonged the survival of lung cancer xenograft mice.

## DISCUSSION

A variety of scenarios, notably smoking and inflammation induce high levels of COX-2/PGE2 [24]. Although we previously reported that binding of rVP1 to integrins induces cancer apoptosis [13] and decreases cancer invasion/metastasis [16], the relationship between rVP-mediated effects and COX-2/PGE2 was unclear. The results of this study demonstrate for the first time that rVP1 is able to suppress COX-2/PGE2-mediated signaling transduction to attenuate cancer invasiveness (Figures 1, 2 and 3). In view of these findings, it might be worthwhile exploring whether the COX-2/PGE2-related adverse effects of smoking and inflammation can be modulated by rVP1.

rVP1 inhibits cancer cell migration/invasion by downregulating integrin/FAK/Akt/GSK-3β signaling and MMP-2 activity [16]. However, it was not clear how interaction of rVP1 with integrin could attenuate Akt/GSK-3β signaling. Here we showed that rVP1 inhibited lung cancer invasion by decreasing FAK/PI3K/PIP3 to downregulate ILK and phospho-Akt^S473^ in the lipid rafts (Figure 2 and 3). ILK is known to regulate not only cell survival and proliferation but also migration by connecting the cytoplasmic domains of β-integrins to the actin cytoskeleton in diverse cell types [22]. Our results, however, demonstrated that rVP1-mediated inhibition of ILK resulted in the downregulation of IKK/NF-κB signaling and COX-2/PGE2 expression (Figure 3C and 3D). Overexpression of ILK or NF-κB p65 blocked the inhibitory effect of rVP1 on COX-2 expression (Figs. 3E) to induce MIG-7, EMT and MMP2 activity as well as invasiveness of lung cancer cells (Supplementary Figure 1). Moreover, overexpression of COX-2 or MIG-7 reversed the inhibitory effects of rVP1 on invasion/migration (Figure 4A and 4B). ILK may thus upregulate COX-2 and MIG-7 to enhance EMT and migration/invasion of cancer cells. Whether rVP1 can regulate the actin cytoskeleton to attenuate COX-2 and MIG-7 or regulate COX-2 and MIG-7 to attenuate the actin cytoskeleton remains to be elucidated.

EMT is enhanced by transcription repressors notably, ZEB1, Twist and Snail that interact with E-box elements located within the proximal region of the E-cadherin (CDH1) promoter to suppress E-cadherin [17, 25]. Phosphorylated prohibitin has been shown to decrease E-cadherin via increasing ZEB1 and Snail but not Twist [15]. In comparison, MIG-7 decreases E-cadherin by increasing ZEB1 and Twist but not Snail [12]; (Supplementary Figure 3). rVP1 suppresses cervical cancer cell metastasis by decreasing membrane bound PIP3 leading to modulation of phospho-PHB^T258^ in the lipid rafts of the cervical cancer cells and inactivation of Raf-1/ERK [15]. Since rVP1 also downregulated MIG-7 whereas overexpression of MIG-7 reversed the inhibitory effects of rVP1 on EMT and invasion (Figure 4A and 4B) it is likely that MIG-7 and phosphorylated prohibitin might have a synergistic or additive effect on E-cadherin, EMT and cell migration/invasion. Whether there is a functional relationship between phospho-PHB^T258^ and MIG-7 and whether rVP1 attenuates either one to affect the other or both via different pathways are currently under investigation.

In summary, our study demonstrates that rVP1 downregulates integrin/FAK/PI3K and PIP3 as well as ILK and phospho-Akt in the raft domains to attenuate IKK/NF-κB signaling and reduce COX-2 and MIG-7 level leading to suppression of EMT and the invasion/metastasis of lung cancer cells *in vitro* and *in vivo*. These results suggest that agents such as rVP1 that selectively modulate COX-2/PGE2 and MIG-7 level may have great potential for development as novel therapeutic agents for metastatic cancer.

## MATERIALS AND METHODS

### Materials

Mouse anti-vimentin (V9) antibodies and recombinant PGE2 were purchased from Sigma-Aldrich (St Louis, MO). Rabbit anti-MIG-7 antibodies were obtained from Abcam (Cambridge, UK). Mouse anti-β-actin, goat anti-COX-2 (M-19), rabbit anti-E-cadherin (H-108), rabbit anti-ZEB1 (H-102), rabbit anti-Snail (H-130) antibodies as well as horseradish peroxidase-conjugated anti-mouse IgG, horseradish peroxidase-conjugated anti-goat IgG and horseradish peroxidase-conjugated anti-rabbit IgG antibodies were purchased from Santa Cruz Biotechnology (Santa Cruz, CA). Rabbit anti-FAK and mouse anti-phospho-FAK^Y397^ antibodies were obtained from BD Biosciences (Bedford, MA). Rabbit-anti-ILK, rabbit anti-phospho-PI3Kp85, rabbit anti-phospho-PI3Kp85^Y458^, rabbit anti-PTEN, rabbit anti-phospho-PTEN^S385^, rabbit anti-Akt, rabbit anti-phospho-Akt ^Ser473^, rabbit anti-phospho-IKKα/β^Ser176/180^, rabbit anti-phospho-NF-κB p65^Ser536^ and rabbit anti-clathrin HC antibodies were obtained from Cell Signaling Technology (Beverly, MA). Mouse anti-integrin-β1 (MAB1965), mouse anti-IKK/, rabbit anti-IκBα and rabbit anti-NF-κB p65 antibodies were obtained from Millipore (Bedford, MA). Rabbit anti-caveolin-1 antibodies were obtained from Upstate Biotechnology (Charlottesville, VA). Phosphatidylinositol (3,4,5)-trisphosphate diC16 (PI(3,4,5)P3 diC16) was purchased from Echelon Biosciences (Salt Lake City, UT). Cell proliferation was determined routinely by WST-1 reagent (Roche Diagnostics, Mannheim, Germany) as described previously [13, 26].

### Human lung cancer cell lines and mice

A549 cell line (ATCC: CCL-185) was maintained in DMEM medium (GibcoBRL Life Technologies, Grand Island, NY) supplemented with 10% fetal bovine serum (FBS; GibcoBRL Life Technologies) and 1% penicillin-streptomycin-neomycin (GibcoBRL Life Technologies). H1299 (ATCC: CRL-5803) and CL1-5 [27] were maintained in RPMI medium (GibcoBRL Life Technologies) supplemented with 10% FBS and 1% penicillin-streptomycin-neomycin. All cells were cultured in a humidified incubator containing 5% CO_2_ at 37°C. Male SCID mice (C.B17/lcr-Prkdc ^scid^/CrlNarl) were purchased from the National Laboratory Animal Center (Taipei, Taiwan). All animal care and *in vivo* experiments were performed in compliance with the guidelines of the Academia Sinica Institutional Animal Care and Utilization Committee. Mice were provided a standard laboratory diet and distilled water and kept on a 12-h light/dark cycle at 25 ± 2°C and 55 ± 5% relative humidity.

### Purification of recombinant VP1 protein

Purification of recombinant VP1 protein was carried out as described previously [28]. In brief, the VP1 gene in the expression vector pET24a(+) (Novagen, Madison, WI) was expressed in *Escherichia coli*. After breaking up the bacteria with a microfluidizer in TEN buffer (50 mM Tris–HCl, pH 8.0, 1 mM EDTA, 0.1 M NaCl), the pellet was washed with 0.5% deoxycholate in TEN buffer, followed by rinsing with TEN buffer and resuspended in binding buffer (20 mM Tris–HCl, pH 8.0, 0.5 M NaCl, 8 M urea). The solution was then applied to a metal-chelating affinity column and eluted with a gradient of 0–0.2 M imidazole. SDS was then added to the protein solution to a final concentration of 1%. The protein solution was subsequently applied to a Superdex 200 column (GE Healthcare, Piscataway, NJ) and eluted with a buffer solution containing 25 mM Tris–HCl, pH 8.0, 1 mM EDTA, 0.1 M NaCl and 0.05% SDS. The fractions containing rVP1 protein were pooled, concentrated and dialyzed against PBS before use.

### Knockdown and overexpression of proteins

Full-length human COX-2 cDNA (NM 000963.2) and human MIG-7 (DQ080207.2) cDNA derived from A549 cells were amplified by using specific primers (Supplementary Table 1) (Sigma-Proligo, St Louis, MO) and subcloned into pcDNA™6/BioEase™-DEST by Gateway cloning technology (Invitrogen, Carlsbad, CA) to generate *pCOX2* and *pMIG7* plasmids. The insert sequences in the plasmids were confirmed by automated DNA sequencing. pUSEamp, pUSEamp-myr-*Akt*1 (dominant active) and pUSEamp-*Akt*1 K179M (dominant negative) were purchased from Upstate Biotechnology. Full-length ILK (BC001554) cDNA and NF-κBp65 (BC110830) cDNA cloned in pCMV-SPORT6 vectors (*pILK* and *pNF-κBp65*) were purchased from Open Biosystems (Huntsville, AL). Control siRNAs, COX2-siRNA and NF-κBp65-siRNA were obtained from Santa Cruz Biotechnology. The plasmids or siRNAs were transfected into cell lines by PolyJet In Vitro DNA Transfection Reagent or GeneMute siRNA and DNA Transfection Reagent (SignaGen laboratories, Ijamsville, MD). MIG-7 siRNA and scrambled control siRNA were purchased from Dharmacon (Thermo Fisher Scientific, Lafayette, CO). The sequence of the MIG-7 siRNA is: GUCGAAGAAAUGAAACUUUUU. MIG-7 siRNA transfection was undertaken using Dharmacon Accell SMARTpool siRNA reagent (Thermo Fisher Scientific) according to the protocol recommended by Dharmacon (Heidelberg, Germany). The transfected cells were used for a variety of experiments as indicated and expression of target proteins in the transfected cells was determined 48 h after transfection, unless specified otherwise.

### Extraction of cellular proteins

Sixty milligrams of frozen tissue was immersed in 2 ml RIPA buffer (Santa Cruz Biotechnology) containing proteinase and phosphatase inhibitor cocktail (Roche, Mannheim, Germany) and then lysed with homogenizer at 4°C. After centrifugation at 14000 × g for 60 min at 4°C, the pellets were discarded and the supernatant containing proteins was stored -80°C until needed. Equal amounts of protein (60 μg) were loaded onto 10% SDS-PAGE and transferred to PVDF membrane (Millipore, Bedford, MA) for western blot (immunoblot) analysis.

### Extraction of membrane raft proteins

Membrane raft proteins were extracted as described previously [29]. Briefly, cells (2 × 10^6^) were washed in ice-cold PBS and lysed by incubation for 30 min in ice-cold lysis buffer (0.5% Triton X-100, 150 mM NaCl, 20 mM Tris–HCl, pH 7.5) containing proteinase and phosphatase inhibitor cocktail (Roche). After centrifugation at 14,000 rpm for 30 min at 4°C, the supernatants (containing the Triton X-100 soluble fractions) were collected and referred to as the cytosolic plus non-raft membrane (C+M) fraction. The insoluble pellets were resuspended in the same lysis buffer supplemented with 0.5% SDS and 2 mM DTT and sonicated for 10 min at 4°C. After centrifugation at 14,000 rpm for 30 min at 4°C, the supernatants consisting of membrane raft proteins were collected and analyzed by immunoblotting.

### Immunoblotting and gelatin zymographic analysis

Immunoblotting and gelatinolytic activities of matrix metalloproteinase-2 (MMP-2) and MMP-9 were performed as described previously [12, 30].

### Migration and invasion assays

*In vitro* migration and invasion assays were performed as described previously [12, 30].

### ELISA for PGE2

The levels of intracellular and secreted PGE2 in the cell lysate and cell-conditioned medium were measured using a commercially available PGE2 EIA assay kit (Amersham, Arlington Heights, IL) according to the manufacturer's instructions.

### ELISA for PIP3

H1299 and CL1-5 cells (1 10^8^) were treated with or without rVP1 for 24 h in 0.5% FBS medium. The cells were washed twice with ice-cold PBS, and phospholipids were extracted and PIP3 levels were measured by ELISA assay using PIP3 Mass ELISA Kit (Echelon Biotechnology) according to the manufacturer's instructions.

### Preparation of pCMV-GFP/luciferase-lentivirus and establishment of stable CL1-5^GL^ cell lines

To generate CL1-5^GL^ cells expressing both green fluorescent protein (GFP) and luciferase in CL1-5 cells, pCMV-GFP/luciferase-lentivirus was prepared as described previously [16]. CL1-5^GL^ cells were then produced by infecting CL1-5 cells with pCMV-GFP/luciferase-lentivirus. Flow cytometric analysis revealed that more than 99% of cells expressed GFP. Cell lysates were also harvested for validation of luciferase activity using a Minilumat LB 9506 luminometer (Berthold, Wildbach, Germany). Luciferase activity was confirmed by measuring the photon counts of serial cell dilutions (1~10^5^) by the IVIS Imaging System (Xenogen, Alameda, CA).

### *In vivo* experimental metastasis assay for rVP1-pretreated cancer cells

Human lung cancer cells (CL1-5^GL^ cells) were pretreated with or without 0.3μM rVP1 in 0.5% FBS-medium at 37°C for 24 h. The cells (1 × 10^6^ cells/50 μl PBS/mouse) were injected into male SCID mice per group via the tail vein. Metastatic progression was monitored weekly and quantified using a noninvasive bioluminescence IVIS Imaging System (Xenogen, Alameda, CA) as described previously [16]. After 5 weeks, three mice were killed for necropsy and the other 8 mice were kept for survival studies. The lungs of the sacrificed mice were isolated, collected and processed for H&E/immunohistochemistry staining. The number of tumor colonies in the lungs was counted, and relative lung weight (lung weight/body weight) was recorded.

### Experimental xenograft murine metastasis assay to test *in vivo* effects of rVP1

Male SCID mice (*n* = 11/group) were implanted with 50 μl RPMI medium (vehicle) or 1 × 10^6^/50 μl tumor cells (CL1-5^GL^) on day 0 by lateral tail vein injection. Metastatic progression was monitored weekly and quantified using a noninvasive bioluminescence IVIS Imaging System (Xenogen, Alameda, CA) as described previously [16]. One week after lateral tail vein injection, one group of mice was treated with vehicle and another group was treated with rVP1 (5 or 15 mg/kg body weight in 100 μl PBS) via tail-vein injection three times per week for 4 weeks. Three mice in each group were killed for necropsy and the other 9 mice were kept for survival studies. The lungs of the sacrificed mice were isolated, collected and processed for H&E/immunohistochemistry staining. The number of tumor colonies in the lungs was counted, and relative lung weight (lung weight/body weight) recorded.

### Immunohistochemistry and histopathology examination

The mouse tissue samples were embedded in paraffin and cut to 4 μm thicknesses. Samples were stained with hematoxylin and eosin (H&E) and immunostained with different antibodies, and examined in the same manner as described previously [16, 30]. The images were scanned into a digital format by Scanscope XT system (Aperio Technologies, Vista, CA) and analyzed using Aperio ImageScope 9.1 software (Aperio Technologies).

### Statistics

All statistical comparisons were made with two-tailed tests. The survival time was assessed using Kaplan-Meier curves and tested for significance by the log-rank test. Statistical evaluation was performed using GraphPad Prism version 5.0 for Microsoft Windows (GraphPad Software, La Jolla, CA). Differences between groups were considered statistically significant at *P < 0.05 or **P < 0.01.

## SUPPLEMENTARY MATERIAL, FIGURES AND TABLE


