# Supplementary material for: Recombinant viral capsid protein VP1 suppresses lung cancer metastasis by inhibiting COX-2/PGE2 and MIG-7
Source: Oncotarget. 2014 May 29;5(11):3931–43. doi: 10.18632/oncotarget.2040 (PMC4116532; doi:10.18632/oncotarget.2040)
Supplement: Supplementary file 1 [file oncotarget-05-3931-s001.pdf]

Recombinant viral capsid protein VP1 suppresses lung cancer metastasis by inhibiting COX-2/PGE2 and MIG-7

Supplementary Material

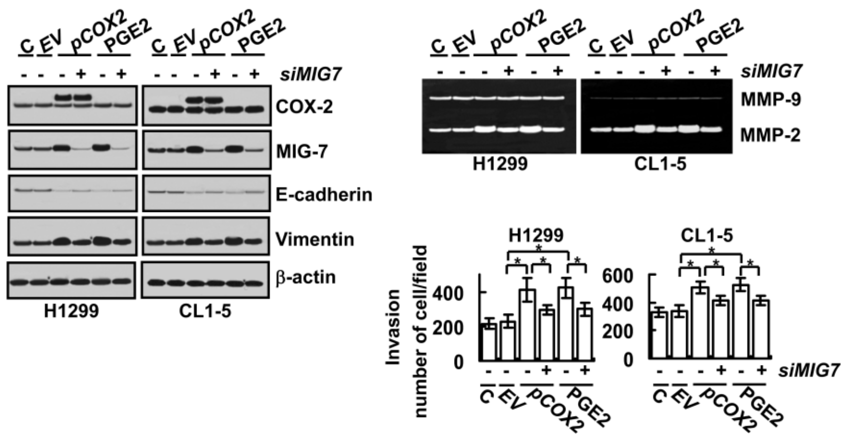

Supplementary Figure 1: MIG-7 protein plays an important role in COX-2/PGE2/EP4-induced migration and invasion of lung cancer cells. Parental lung cancer cells (H1299 and CL1-5; C) were transfected with empty vector (EV), pCOX-2 plasmids (*pCOX2*), control scrambled siRNAs (-*siMIG7*) or MIG7 siRNAs (+*siMIG7*) for 48 h or treated with PGE2 (20 μg/ml) for 24 h as indicated. Cells were examined by immunoblotting for protein expression, zymography for MMP activity and transwell assay for cell invasion as described in Materials and Methods. Blots are representative of three independent experiments. Data represent means ± SD of three independent experiments; $^{*}P < 0.05$ by t-test.

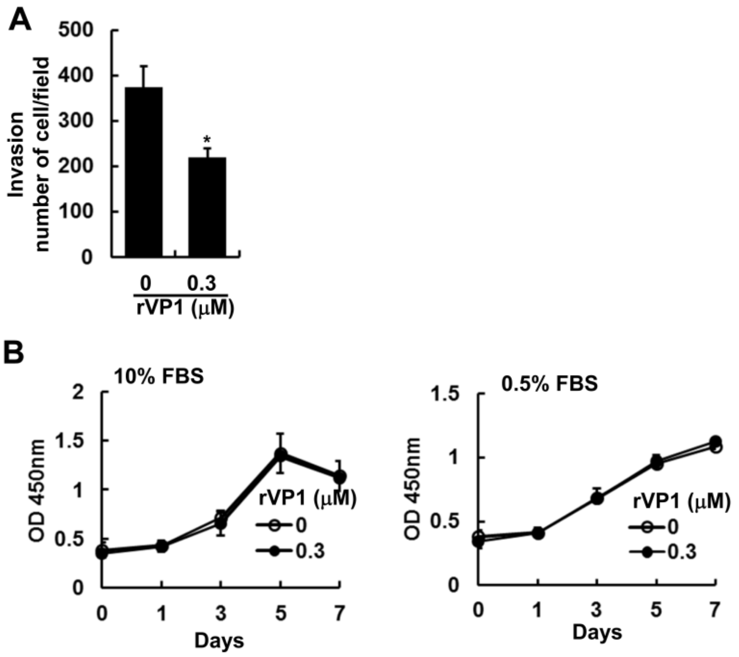

Supplementary Figure 2: Treatment of CL1-5^{GL} lung cancer cells with low concentration of rVP1 decreases invasion but not growth of the cells. CL1-5^{GL} cells were pretreated with or without 0.3 μM rVP1 in 0.5% FBS medium as indicated for 24 h. (A) The invasion capability of the cells was measured by transwell migration assay. Data represent means ± SD of three independent experiments; $^{*}P < 0.05$ by t-test. (B) The cells were then seeded in a 96 well-plate (3×10^3 cells/100 μl/well) containing culture medium supplemented with 10% FBS (left panel) or 0.5% FBS (right panel) and cell proliferation was measured on different days using WST-1 assay. Data represent means ± SD of three independent experiments.

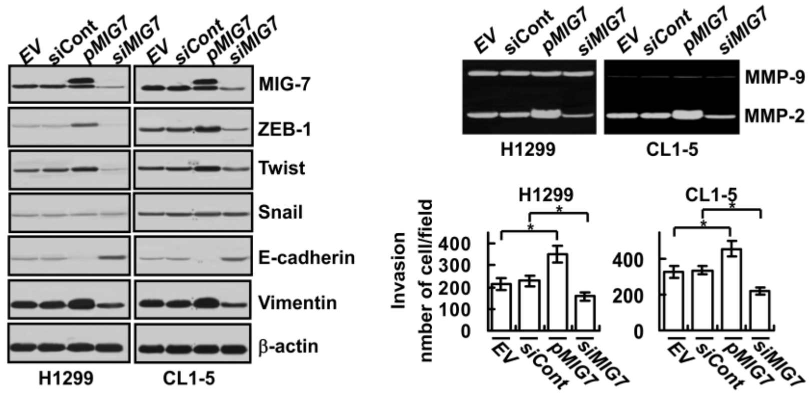

Supplementary Figure 3: MIG-7 protein increases ZEB-1 and Twist but not Snail to enhance EMT, MMP-2 and invasion of lung cancer cells. Lung cancer cells (H1299 and CL1-5) were transfected with empty vector (EV), pMIG-7 plasmids (*pMIG7*), control scrambled siRNAs (*siCont*) or MIG7 siRNAs (*siMIG7*) as indicated for 48 h. Cells were examined by immunoblotting for protein expression, zymography for MMP activity and transwell assay for cell invasion as described in Materials and Methods. Blots are representative of three independent experiments. Data represent means ± SD of three independent experiments; $^{*}P < 0.05$; by t-test.

Supplementary Table 1: Sequences of primer pairs

	Forward primer/reverse primer
MIG-7 (construction)	5′-GGGGACAAGTTTGTACAAAAAAGCAGGCTTCATGGCAGCAAGTAGATGCTCTGGT-3′<br>5′-GGGGACCACTTTGTACAAGAAAGCTGGGTCCTAGGCCGTGATGAATCATGTGACGCA-3′
COX-2 (construction)	5′-GGGGACAAGTTTGTACAAAAAAGCAGGCTTCATGCTCGCCCGCGCCCTGCTGCTG-3′<br>5′-GGGGACCACTTTGTACAAGAAAGCTGGGTCCTACAGTTCAGTCGAACGTTCTTTTAG-3′
